# Dynamic Impact Surface Damage Analysis of 3D Woven Para-Aramid Armour Panels Using NDI Technique

**DOI:** 10.3390/polym13060877

**Published:** 2021-03-12

**Authors:** Mulat Alubel Abtew, Francois Boussu, Pascal Bruniaux, Yan Hong

**Affiliations:** 1 College of Textile and Clothing Engineering, Soochow University, 178 G.J.D. Road, Suzhou 215021, China; 2 Ethiopian Institute of Textile & Fashion Technology, Bahir Dar University, P.O. Box 1037 Bahir Dar, Ethiopia; 3 ENSAIT-GEMTEX Lab, Lille Université, 2 Allée Louise et Victor Champier, 59056 Roubaix, France; francois.boussu@ensait.fr (F.B.); pascal.bruniaux@ensait.fr (P.B.)

**Keywords:** dynamic impact, nondestructive investigations (NDI), impact damage, surface displacement, protective armour, 3D woven panel, high-performance para-aramid fibre

## Abstract

The effects of the yarn composition system inside 3D woven high-performance textiles are not well investigated and understood against their final ballistic impact behaviour. The current study aims to examine the ballistic impact performances of armour panels made of different 3D woven fabric variants through postmortem observations. Four high-performance five-layer 3D woven fabric variants were engineered based on their different warp yarn compositions but similar area density. A 50 × 50 cm^2^ armour system of each variant, which comprises eight nonbonded but aligned panels, namely, 3D-40-8/0 (or 8/0), 3D-40-8/4 (or 8/4), 3D-40-8/8 (or 8/8) and 3D-40-4/8 (or 4/8), were prepared and moulded to resemble female frontal morphology. The armour systems were then tested with nonperforation ballistic impacts according to the National Institute of Justice (NIJ) 0101.06 standard Level-IIIA. Two high-speed cameras were used to capture the event throughout the test. Nondestructive investigation (NDI) using optical microscopic and stereoscopic 3D digital images were employed for the analysis. The armour panels made of the 8/0 and 4/8 fabric variants were perforated, whereas the armour made of the 8/8 and 8/4 fabric variants showed no perforation. Besides, the armour made of the 8/4 fabric variant revealed higher local and global surface displacements than the other armours. The current research findings are useful for further engineering of 3D woven fabric for seamless women’s impact protective clothing.

## 1. Introduction

While developing a body armour system, it should possess various aspects including having good ballistic protection, being reasonably light in weight, flexible, and comfortable [1]. Various parameters for material development [2,3,4], armour panel design [5,6,7,8,9] and its finishing [10] can be considered to achieve such performance. Considering textile material development, its overall performance could be enhanced by various internal factors such as fibre types, yarn properties, material areal density, target ply numbers, target ply sequence etc., [11,12,13]. Besides, the type of fabric and its architecture through various design aspects has also brought about a great impact on the performance and comfort of the intended armour system [14,15,16,17]. Mostly, two-dimensional (2D) woven fabrics and unidirectional (UD) textile laminates made with high-performance fibres (Twaron^®^, Kevlar^®^, Dyneema^®^, and Spectra^®^) are used in soft body armour due to their high resistance-to-impact damage, high strength and lightweight characteristics [18,19,20,21,22]. Besides, such fabric types possess an excellent mechanical properties along with better fatigue life [23]. However, researchers are still continuously investigating the different factors of ballistic textile materials to further improve the overall performance of body armour systems, which could be used in various ballistic protection levels. For example, hybrid panels with different textile materials and arrangements were used to develop a body armour system for better ballistic performance [24,25,26,27]. Recent research also studied the role of interlayer bonding on the kinetic energy absorption capabilities of a multilayer armour system [28]. The effects of various woven fabric architectures (plain, basket, twill and satin) have been investigated against high-speed impact testing to improve the impact resistance performance of armour systems [29]. Different constructional methods in the armour system using woven and cross-ply laminates were also involved and investigated to assess its effect on protection performance [30]. Meanwhile, the involvement of common clothing fabrics as intermediate targets on the penetration of shotgun shell pellets, using ordnance gelatin to simulate soft tissue and thin cowhide to simulate skin, has been also investigated for enhancing the armour system [31]. Various researchers have also intensively studied the surface modifications of textile materials and their effect on the ballistic performance of the armour system [32,33,34,35].

Apart from the commonly used 2D and UD textile structures, 3D woven fabrics are becoming a promising ballistic textile structure due to their excellent moulding ability [36,37,38,39,40] and ballistic protection performance [41,42]. The 3D woven fabric’s structure could be involved not only in planer but also nonplanar body armour systems considering various structural parameters to give better ballistic protection and comfort to the wearer [43]. Thus, some research studies have designed, manufactured and investigated 3D warp interlock fabric structures for the development of a seamless female frontal body armour system. A study has introduced mathematical modelling to develop a seamless armour system for women [44] and used 3D angle-interlock woven fabrics to accommodate the body with better fit and improved protections [8]. Our previous study has investigated the ballistic performances of 3D warp interlock fabrics as compared to their counterpart 2D structures in terms of the back face signature (BFS), the panels’ energy absorbing capability and surface damages while testing against NIJ standard Leve lIIA [45,46]. The result shows that the 3D woven fabric revealed better formability but less ballistic performance as compared to 2D woven fabrics for seamless women’s body armour design. The local panel surface failure modes of the armour panels made of 2D woven and 3D warp interlock different fabric panels (orthogonal layer to layer) were also anlysed. Considering this result, another research involved engineering and manufacturing a new different architecture of 3D woven variants considering warp yarn ratios to enhance their ballistic performance without compromising their moldability [47]. The result shows that the ballistic performances of the 3D angle-interlock woven fabrics were improved in terms of energy absorbation and back face signature through involving an optimum warp yarn compositions system inside the structure. Besides, the overall ballistic performances of the armour systems made of 3D woven fabrics could be investigated and quantified through various methods [48]. A study has employed both experimental tests and finite element simulations to study the ballistic impact damages of the 3D orthogonal woven fabric (3DOWF). The numerical simulation revealed precise impact damage morphologies and residual projectile velocities analysis as compared to experimental values [49]. Another study has also studied the failure mechanisms of the hybrid 3D woven orthogonal having an asymmetric distribution of fibres by using a combination of impact tests and X-ray computed tomography analysis of the failure mechanisms [50]. Another study has also studied the performance of armour panels made with 3D woven and 2D woven fabrics through the postmortem analyses [51]. Similarly, a new nickel–chromium (NiCr) wire technique accompanied by other methods (X-rays, high-speed video, and ultra-high-speed photography) was developed and then introduced to measure yarn displacements in a fabric subjected to ballistic impact [52,53]. Another research work also investigated the effects of global and local modes on the failure process of 3D interlock woven fabric. Even though both modes are significant on the ballistic limit, global localization affects the deformation of the whole fabric through the primary weft yarn pulled out mainly near the free edge of the fabric, whereas the impact location decides the failure mechanism of the primary weft and warp yarns around impact location [54]. The damage mechanisms of the 3D interlock woven fabric were also investigated using a numerical model and validated with an experimental test against ballistic impact [55]. Moreover, the body armour system undergoes very large displacement rates over very short periods during impact and consequently, the panel materials face elastic deformation before plastic deformation. During this high level of deformation, three-dimensional digital image correlation (3D DIC) is an effective method to capture various data [56]. Such methods are usually used to measure the back-face deflection and displacement response on armour panels subjected to ballistic impacts from both small arms fire and bird-strike [57]. Another study also used high-speed digital photography to capture the impact phenomenon of a composite’s back surface to provide basic knowledge of the impact event such as deformation, displacements, residual displacements, damage threshold load, transverse matrix crack initiation and propagation [58].

The main aim of the current paper is to investigate the effect of warp yarn systems on the armour panel damage mechanism, surface displacement, debris deformation and armour panel penetration of armour systems made of 3D woven fabric variants. Experimental methods using a nonperforation standard ballistic impact test equipped with two very high-speed cameras to capture perfectly the stereodigital image, and later optical microscopes were employed to analyse the frontal panel postmortem damage mechanism. The presented research in this paper aims to provide a deeper understanding of the armour panel damage mechanism, surface displacement, postimpact debris deformation and penetration of the armour panel made of 3D woven fabric variants, which may later ultimately be used to guide the design of 3D textiles in body armour systems.

## 2. Materials and Testing Methods

### 2.1. Materials and Armour Panel Target Preparations

**Figure 1:**
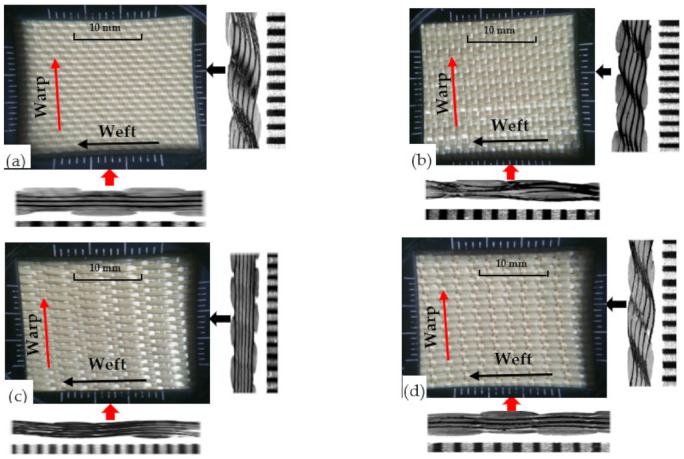
Top view and side sectional micrographs of the different 3D warp interlock variants (**a**) 8/0, (**b**) 8/4, (**c**) 8/8, and (**d**) 4/8.

**Figure 2:**
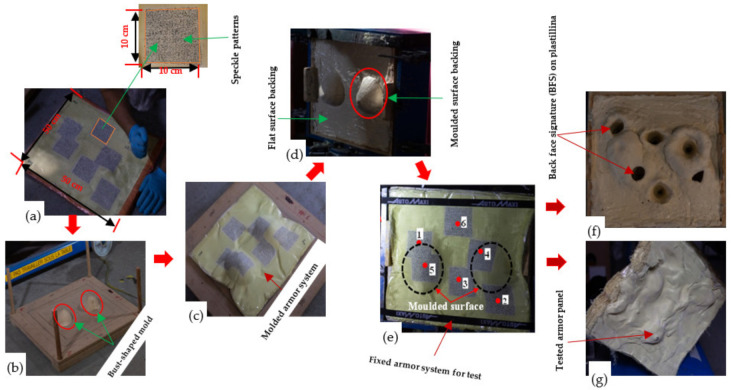
Armour panel system preparation before testing and after testing: (**a**) nondeformed aligned armour system with random speckle pattern; (**b**) customized forming equipment; (**c**) molded armour panels to resemble frontal women morphology; (**d**) prepared backing material (plastillina) with inverted women frontal form; (**e**) armour panels ready for the test based on NIJ-Level IIIA; (**f**) backing panels with deformation after the test and (**g**) back view of the tested armour system.

Four variants of 3D warp interlock orthogonal Layer-to-Layer (O-L) fabric based on different warp yarn ratio systems were designed and manufactured Figure 1. In particular, the fabric structure variants were designed using TexGen^®^ software by considering specific binding and stuffer warp yarn compositions/ratios (Binding: Stuffer) such as 8/0 (Figure 1a), 8/4 (Figure 1b), 8/8 (Figure 1c), and 4/8 (Figure 1d) within the weave repeat unit of each fabric variant structure. This means that, for example, in fabric with variant 8:4, in a single repeat unit (total 12 warp yarn), there are 8 binder warp yarns and 4 stuffer warp yarns. All the fabric variants were manufactured from the same p-aramid yarn, Twaron^®^, having 930dtex linear density, 2.35 mN/tex tenacity, 225 N strength, and 3.45% elongation at break, purchased from Teijin Aramid, a subsidiary of the Teijin Group, The Netherland. Besides, the fabric variants were also manufactured with similar weft yarn layers (five layers), yarn densities (48 warps/cm/panel and 50 wefts/cm/panel) and total theoretical fabric weight (970 gm/m^2^). The utilized high-performance p-aramid yarn (Twaron^®^) was twisted at 25 twists per metre (TPM) throughout the fibre before fabric production. Each fabric was exclusively manufactured in the semiautomatic weaving loom which was equipped with 24 warp beams. However, due to the different interlinking depth of the warp yarn through the weft layers, the average thickness of each fabric’s variants was found to be different (1.42, 1.44, 1.52, and 1.63 mm for variants 8/0, 8/4, 8/8, and 4/8, respectively). The parameter, design and manufacturing processes of the fabrics were also discussed in detail in our previous research work [59]. Four armour panel systems having surface areas of 50 × 50 cm^2^ and comprising nonbonded but angled 8-panels (40 layers) from each 3D warp interlock variant were prepared (Figure 2a). However, the armour systems were firmly taped together at the four-edge using scotch tape to avoid the fibre unravelling and the layers slippage from their original positions. For a better recording of the ballistic phenomenon with a high-speed camera, a very dense and random speckle pattern on the 10 cm × 10 cm area around the selected six-shot points was also systematically drawn on the strike faces of the armour panel. The armour system was then moulded to resemble the female frontal shape using a customized forming bench as shown in Figure 2b.

The backing material (plastillina) box was chosen based on NIJ standard recommendations and prepared with the flat inverted form of a bust-shaped mould (Figure 2c) to accommodate the formed armour system (Figure 2d) and fixed to the backing with the narrow fabrics on the four edges (Figure 2e). It was then firmly attached to the mainframe before ballistic impact testing. After the test, the required data to investigate the different ballistic performance analysis could be then extracted from the backing materials and tested armour panel as shown in Figure 2f,g respectively.

### 2.2. Ballistic Testing Methods and Procedures

In our testing campaign, unlike in perforating ballasting testing, we employed nonperforation ballistic testing to investigate the postmortem analysis of the armour panels made with the different 3D woven fabric variants. The test was carried out according to NIJ Standard-0101.06 Level IIIA using 9 mm × 19 mm Full Metal Jacketed Round Nose (FMJ RN) and 8.0 gm bullets with a velocity of 426 ± 9 m/s at six shots per armour panel system in specified areas [60].

**Figure 3:**
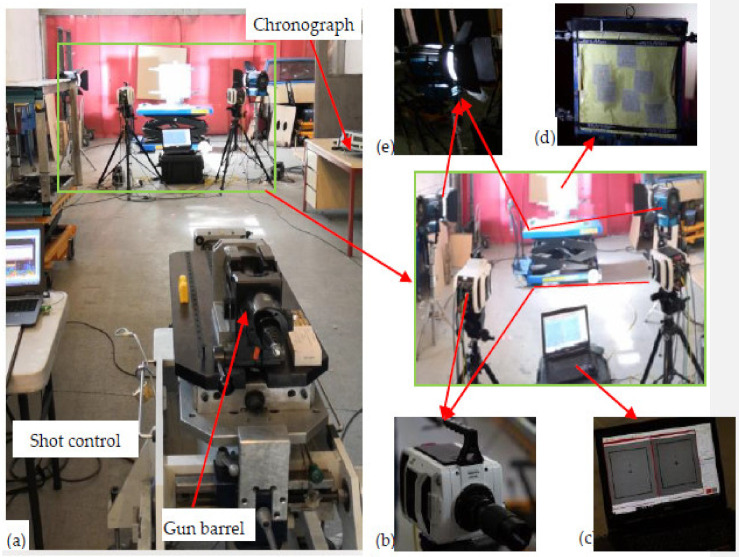
Ballistic testing facility: (**a**) the whole set-up, (**b**) high-speed camera, (**c**) computer system to synchronize the bullet and the high-speed camera recording, (**d**) armour panel system for testing and (**e**) high-voltage lighting system.

Figure 3 shows the ballistic testing equipment and its set up for testing. The nondestructive test (NDT) using microscopic and stereodigital (stereo-DIC) images were employed to analyse the armour postmortem phenomena such as debris core deformations, the number of responsible panels, surface damage and displacements of the armour. While undertaking the panel surface analysis, in case of using the last panels deformations, we examined the backs of the first panels. This is because it was not possible to find the last panel deformation with the same condition for all armour systems. For this reason, two Phantom V1212 very high-speed cameras with 56,000 fps, 384 × 400 pixels, and 280 mm focal length were installed on the right and left sides at 45° angle from the shot axis line to record the whole ballistic impact event. These cameras allowed us to observe the whole impacted process, mainly around the front local impact area. Besides, the two cameras were coordinated along with the shot timing to properly record the impact events. In general, the recording was set to start at 0.018 ms, before the bullet touched the armour, and stopped after recording after a total time of approximately 3.6 ms. The two-camera video was then synchronized together to give the stereoscopic video. The data recorded by the high-speed camera were processed by the TEMA with track eye motion analysis software and then exported in the form of stereoscopic images. This high-speed 3D photograph at a fraction of a microsecond frame, which greatly helps to study in detail the dynamic response of the armour panel and visual surface displacement analysis. Besides, a 450-Watt lighting system with constant light output and synchronized to the signal of the high-speed cameras was used.

Each of the moulded armour panel systems was then supported by the backing materials (plastillina) box and later firmly attached to the mainframe before every shot. The armour panels were set at a distance of 10 m from the nose of a 280 mm longer gun barrel before the test. Doppler radar system (5 m position) and chronograph (9 m position) were used to capture the projectile velocity. So that the backing material resembled the human body, it was kept in the heating oven with an average value of 38.3 °C during the first (1st) shot and 35.3 °C during the last (6th). The ballistic tests were conducted at the Centre de Recherche’et d’expertise De La Logistique (CREL) of France. An optical microscope and digital camera were used to investigate the panel damage properties both at the microscale and macroscale level.

## 3. Results and Discussions

In this section, the surface damage and its 3D visual displacement, projectile deformation and panel penetration systems of armour panels made of 3D woven fabric variants with different warp yarn ratios will be discussed. The stereoscopic images captured by high-speed cameras were used to analyse the 3D visual surface displacements of the armour system at different impact timing. The optical microscope and digital cameras were also involved in the analysis of the different panel penetration and panel surface damage of the armour system.

### 3.1. Postmortem Analysis on Impacted Panels and Projectiles

#### 3.1.1. Panels in Armour System Responsible to Halt Projectile

**Figure 4:**
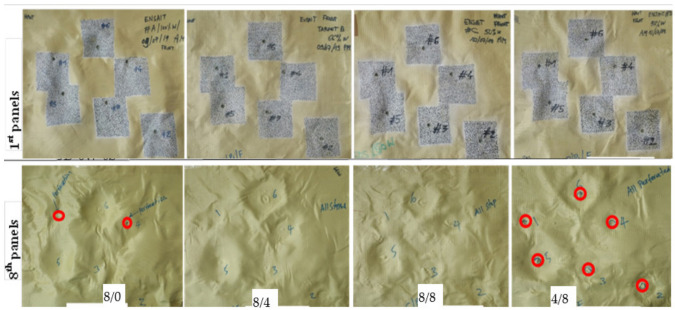
The striking faces of the 1st and 8th (last) panel of the tested armour systems with various shot points.

The number of panels at which the bullet stopped while testing the 3D warp interlocks fabrics considering various shot points was computed. Regardless of the penetration level of the bullet, the number of panels where the bullet trapped was considered for better understanding and comparisons. The armour system was made of the four variants of the 3D warp interlock fabrics which are made of different bindings: the stuffer ratios were considered for analysis. However, since armour system 4/8 was penetrated in all shot points and armour system 8/0 in two shots (one and four shot points), the values in the specific positions were excluded while discussing the projectile arresting panels and their postmortem analysis. Figure 4 shows the pictures of the striking faces of the 1st and 8th panels of the different armour systems at various shot points.

**Figure 5:**
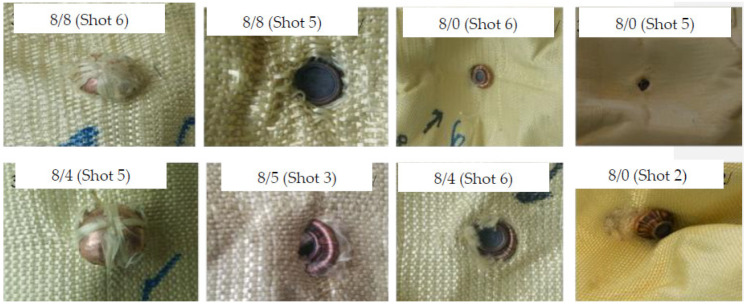
Some trapped projectiles inside the armour panel system.

The different trapped projectiles were also carefully collected, and their length and diameter measured for the postmortem analysis of projectile deformations. Some of the projectiles trapped at various shot points by different panels are shown in Figure 5.

**Figure 6:**
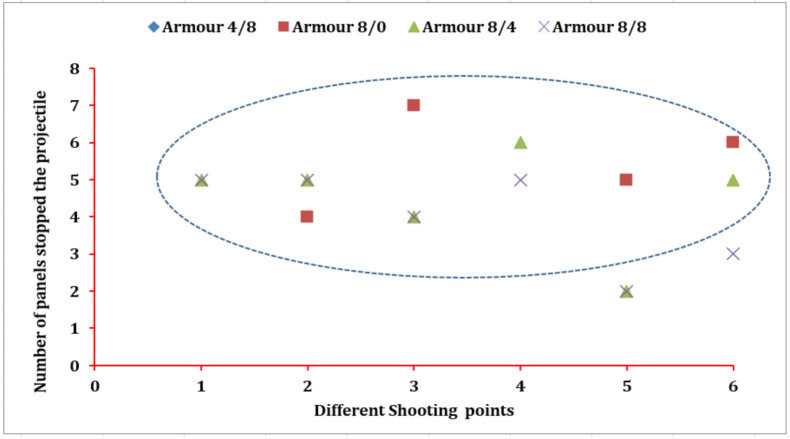
Armour panel number responsible to stop the projectile (All shots penetrated armour system 4/8, and shots one and four penetrated armour system 8/0).

In this section, the number of panels involved to stop the projectile by the different armour systems at the different shot points will be also discussed. Figure 6 shows the responsible number of panels in the armour systems made of different 3D warp interlock fabric variants to arrest the projectile at the designated shot points. Based on the results, armour systems 8/4 and 8/8 revealed the same number of panels responsible to halt the projectile in the majority of the shot points. For example, those armour systems involved 5 panels at shot points 1 and 2 to arrest the projectile.

On the other hand, panels 4 and 2 were accountable for arresting the projectile in the mentioned two armour systems at shot points 3 and 5, respectively. Armour system 8/8 uses a lesser number of panels to stop the projectile at shot points 4 and 6 as compared to the armour panels of 8/4. Besides, the armour panels of variant 8/0, except shot point two, involved a higher number of fabric panels in the projectile arresting mechanism than the armour systems of 8/4 and 8/8.

Without considering the whole penetrated armour panels, 4/8, and 8/0 at shot points 1 and 4, the armour panel made of 8/0 variants recorded the highest number of panels (seven panels) stopping the projectile at shot point three as compared to all other armour systems. On the contrary, the armour panels of 8/4 and 8/8 contributed the minimum number of panels (2) to halt the impacted projectile at shot point 5. However, the number of panels required by the majority of armour systems to stop the projectiles at different shot points located between 4 to 6 panels. (In this investigation, even if it is not very significant, the moulding process also affected the ballistic resistance of the armour system. As discussed in the previous chapter, this might be due to both the reduction of the areal density of the fabric and some yarn failures at the moulded area.) In conclusion, the different warp yarn interchange ratio inside the 3D warp interlock structure greatly influences the number of panels required in the armour systems to stop the projectiles regardless of the shot points. The more balanced the proportions of the binder and stuffer warp yarn inside the warp interlock fabric, the better ballistic performance with a smaller number of panel penetration was achieved. For example, an armour system with 50% of each binding and stuffer warp yarn, 8/8, revealed a minimum number of panels required to halt the projectile followed by armour system 8/4 in the majority of shot points. On the contrary, an armour system with less balanced warp yarn proportions, 4/8, and 8/0, needs a higher number of panels to resist the projectile.

#### 3.1.2. Failure Mechanisms–Amour Panels Based on Different Variants

During ballistic testing, the tested armour panel faces a specific type of shock due to projectile kinetic energy. Meanwhile, the textile-based armour also generates self-resistance toward the bullet impact. In the nonperforation ballistic impact process, generally, some of the kinetic energy could be absorbed by the armour target while the other is transmitted beyond the panel to the backing material (plastillina). During this phenomenon, the propagated shock energy wave on the panels could cause different local and global surface damages such as target compression below the projectile and around the impacted zone, cone formation and primary yarn failure. Besides, the bowing of the yarn, and the friction between the projectile and the target also occurred depending on various parameters. In general, the energy absorbed by the armour target could be performed by various kinds of energy-absorbing mechanism including yarn pullout, surface damage and energy-absorbing mechanisms. Generally, the last panels of the ballistic material for the perforation-ballistic testing will be considered for failure mechanisms analysis. However, in the nonperforation ballistic testing, the panels are not likely to be penetrated and projectiles are halted in different layers of the panels.

**Figure 7:**
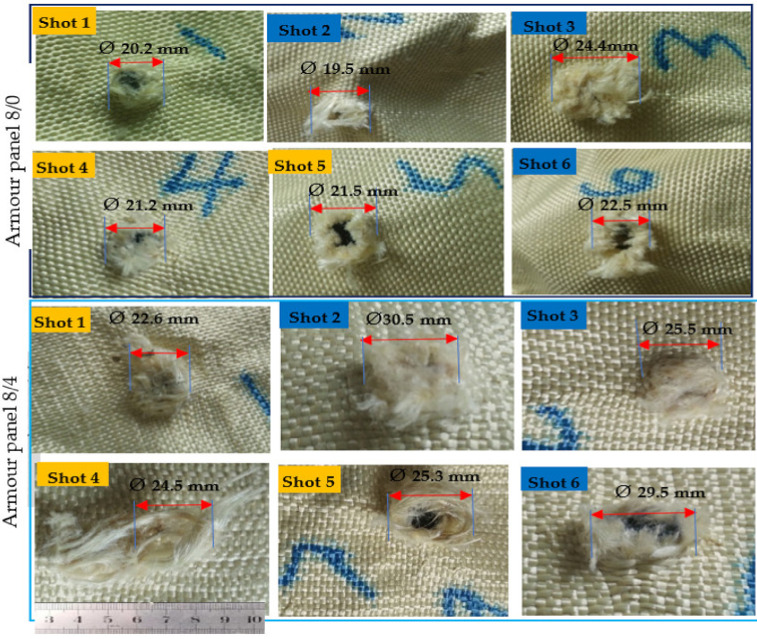
Typical impact surface damage and its diameter of armour panels made of fabric variants 8/0 and 8/4 at moulded (shot 1, 4 and 5) and nonmoulded areas (shot 2, 3 and 6).

**Figure 8:**
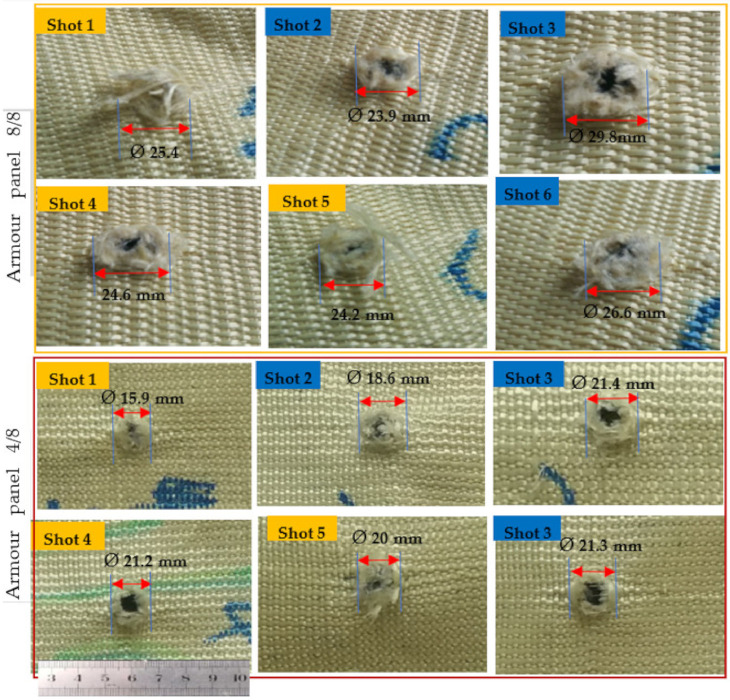
Typical impact surface damage and its maximum diameter of armour panels made of fabric variants 8/8 and 4/8 at moulded (shot 1, 4 and 5) and nonmoulded areas (shot 2, 3 and 6).

This makes it very difficult to have an analysis of the final layers for different armour systems. Considering this, in this section, we have discussed the different surface failure properties and damaged mechanisms at the back sides of the first panels of the armour system made with different 3D warp interlock fabric variants. Based on the test results, in general, the armour panels of 8/0 and 4/8 revealed very sharp and narrow damage diameters with minimum values for all shot points at both moulded (shot 1, 4 and 5) and nonmoulded (shot 2, 3 and 6) areas. For example, Figure 7 and Figure 8 show the impact surface damage mechanisms of the armour panel fabric variants (8/0 and 8/4) and (8/8 and 4/8), respectively, at the moulded (shot 1, 4 and 5) and nonmoulded areas (shot 2, 3 and 6).

On the contrary, the armour panels of armour systems 8/0 and 4/8 suffer more severe damage and prominently larger diameter after penetration. This indicated that the involvement of both the primary and secondary yarns in the ballistic impact process for earlier armour panels, 8/0 and 4/8, were much higher than the later one, armour panels 8/0 and 4/8, as shown both in Figure 7 and Figure 8. This is because the different fabric properties of the 3D warp interlock fabric variants including fabric architecture and warp yarn compositions inside the structure play a great role in the performance. Such involvements of higher yarn in the impact process also give better energy absorption capabilities for the mentioned armour systems [47]. Besides, apart from the involvements of the different fabric variants in armour system, the condition of the impacted area (moulded or nonmoulded) has also shown a great influence on the capability of the armour to stop the projectile with different damage surface mechanisms of the armour panels. In general, the moulded areas recorded lower damage diameters as compared to the nonmoulded areas of the armour panel for the majority of the shot areas. This is due to the fact that the forming process could reduce the surface areal density of the armour panel fabric in the specified region. Moreover, the forming force during the process also creates not only straightening and higher yarn tension but also some yarn failurity modes at the moulded area.

Such yarn straightening and higher tension might causes the opening of the crimp and then allows the projectile to easily penetrate into the armour panel. Based on this, the average damage diameters of moulded and nonmoulded areas for armour panels 8/0 were Ø20.98 and Ø22.13 mm, respectively. Besides, 8/4, 8/8, 4/8 recorded an average damage diameter of Ø24.13, Ø24.06 and Ø19.03 mm, respectively, at the moulded, and Ø28.5, Ø26.76 and Ø20.43 at the nonmoulded areas, respectively.

Regarding the surface failure, armour panels 8/8 and 8/4 faced both fibrillation (splitting of the yarns) and yarn pull out in the majority of its shot areas to absorb the projectile impact energy. In the formation of such fibrillation, those panels could absorb more kinetic energy during projectile penetration. Besides, the uncrimping of the binding yarns in these armour panels also helps to absorb higher impact energy. Such failures are normally created by the fatigue and abrasion action of the ballistic projectile while pushing and penetrating the fabrics, and this panel faces more severe yarn damage and fibrillations through its transverse length direction as the projectile penetrates. On the contrary, armour panels 8/0 and 4/8 possess less fibrillation and yarn pull out rather than the splitting and rupturing of the yarns while projectiles pass the target. This is due to their loose fabric structure and higher yarn mobility at the cross-point of the structure. Such a damage mechanism allows the bullet to easily penetrate the primary yarns in the zone without any tensioning or wrinkle formations.

#### 3.1.3. Postmortem Analysis of Projectile Deformations

**Figure 9:**
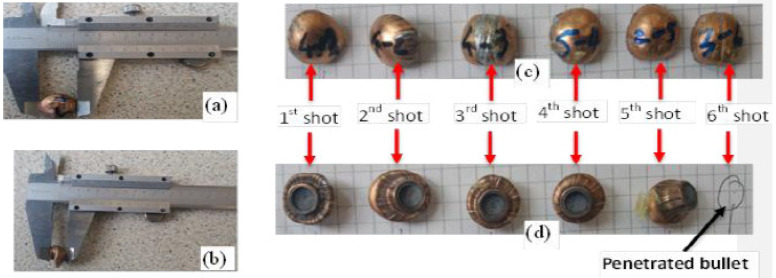
Recovered projectile debris diameter and length measurements (**a**,**b**) measurement system (**c**,**d**). Images of the recovered projectile debris after armour panel test.

In this section, the trapped projectile debris deformation and its mass at different shot points of the armour panels will be measured and analyzed. In the current ballistic test, the trapped debris from four armour systems made of the 3D warp interlock fabrics made with different warp yarn interchange ratios was considered. A total of thirty shots, six shots for every armour panel, were used according to NIJ standard. However, among the thirty projectiles, a total of nine projectiles (six projectiles from armour panel 4/8 and two projectiles from armour panel 8/0) were not considered due to their penetrating throughout the panel. The rest of the twenty-one pieces of debris were measured after the ballistic test using a precise Vernier scale. Some of the shapes and measurements of the debris after impact are shown in Figure 9.

**Figure 10:**
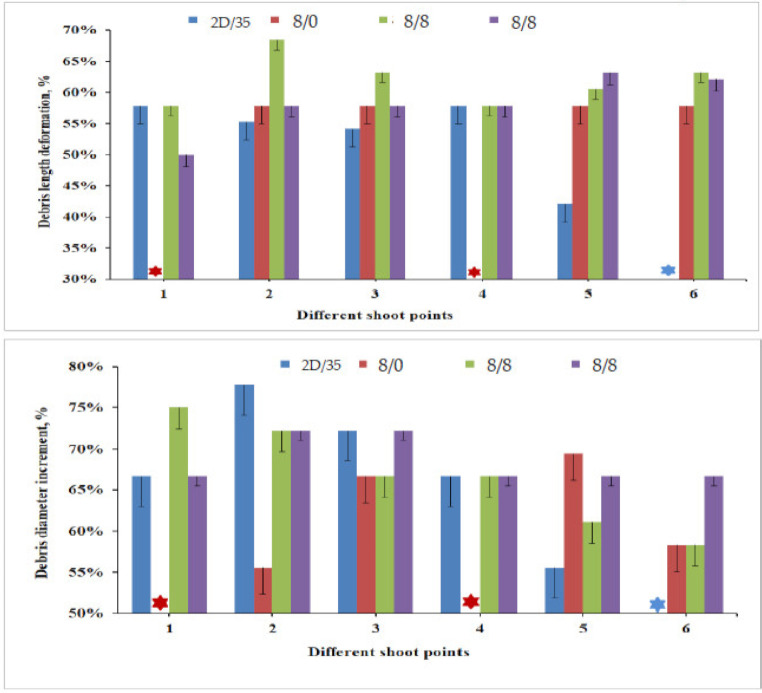
The residual projectile debris deformational percentage for armour panels at various shot points. All shots penetrated for armour panel (4/8), armour panel (8/0) penetrated at shots 1 and 4 (*)).

Unlike the debris measurement values, its deformational percentages both in the length and diameter direction give a better comparison of the different armour systems at various shot points. Figure 10 presents the deformational percentages of the debris length and diameter, respectively, against the original projectile measurement for the different armour systems. In general, unlike the debris length, the diameter of the debris possesses an increment compared to the original projectile measurement values. Based on the result, the projectile impacted onto armour panel 8/4 panels, except shot five, faced much higher longitudinal deformation compared to the other armour system panels at the majority of impact points. Besides, armour panel 8/4 also deformed the projectile with the maximum longitudinal deformation percentage (68%) at shot point two than the other impacted projectiles. Unlike its penetration in shot point one, armour panel 8/0 shows a similar projectile deformational percentage (%) at shot points two and three, but possesses lower values in shot points five and six compared to armour 8/8. In general, projectile deformation values were random and there was not a clear indication that this was influenced by the warp yarn interchange ratio inside the 3D warp interlock fabrics. However, an armour system made of the 3D warp interlock fabrics with a more balanced warp yarn interchange ratio shows better projectile deformation than an armour system made of the 2D plain weave fabrics. The location of the impact shows no significant effect on the postimpact projectile deformations of the armour systems.

#### 3.1.4. Panel Surfaces Displacement at Global and Local Areas

**Figure 11:**
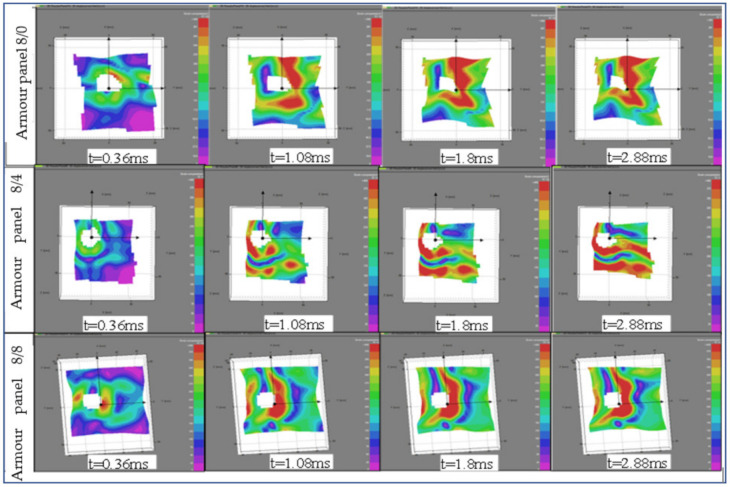
Image sequences of an impacted panel at front with selected time at shot point 3.

The displacements of the surface of the armour panel layer during the ballistic impact is very important not only to understand the armour material’s responses and its energy distribution on the surface but also help to improve its overall performance. Using the two-camera video, it was possible to generate a huge number of images for specific timings; however, only some of the stereoscopic digital images with the real fabric surface image were selected to unveil and analyse the local and global displacement and failure of the different armour panels. We also excluded the armour panel 4/8 since it was fully penetrated by the projectile in all the shot points. Moreover, shot points one, three and five were primarily considered in our image analysis to better understand the postmortem analysis. Figure 11 shows the displacement image sequences of the impacted panel, armour panels 8/0, 8/4 and 8/8, with selected time at shot point 3. In general, the presented panel shows different surface displacements but almost similar panel penetration sizes throughout the events at the given shot points. Based on the observations, armour system 8/4 still involved predominantly the primary yarns at 0.36 ms after impact. On the contrary, considering the same after impact time (0.36 ms), both armour panels 8/0 and 8/8 involved the primary and secondary regions to resist the ballistic impacts. However, the displacement propagations were still accumulated around the local impact regions. Of course, as the after-impact time went further, all the tested armour systems involved both the primary and secondary yarns.

Interestingly, similar trends of the wave propagations were observed within the same armour system panels as time after impact went further from 0.36 ms to 2.88 ms. However, the wave propagation trends and their effect on the panels’ surface displacements were realized in different directions among the armour systems. For example, the wave propagations in the panel surface of armour panel 8/0 seem well concentrated in certain areas near the local impact points and contours of the penetrated panel. Besides, the local displacements were found well distributed both in the weft (horizontal) and warp (vertical) directions. On the contrary, the higher surface displacement by the wave propagations of armour panel 8/4 through time was distributed in different regions both near to and away from the impact points. The high surface displacements of this panel were seen along the weft (horizontal) directions. As shown in Figure 11, armour panel 8/8 also shows its own high displacement value location and direction. In this panel, similar to armour panel 8/0, the high strain was more concentrated around the local impact point but propagated with different intensities in the warp direction (vertical) as time passed.

**Figure 12:**
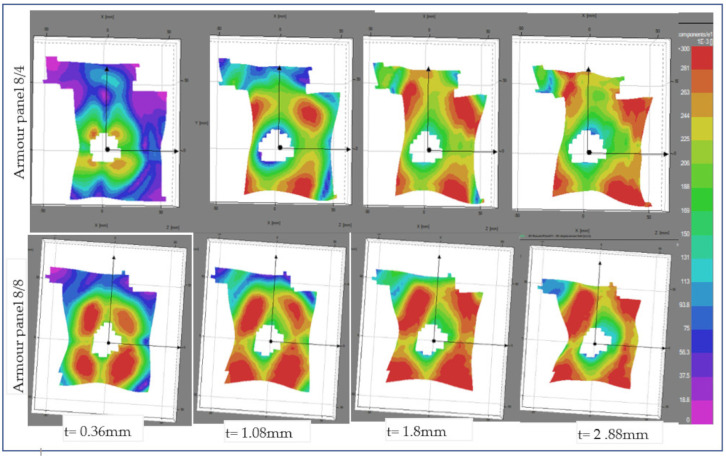
Frontal stereoscopic images sequences of an impacted armour panel selected impact timing at shot 5.

The different images of the impacted panels for the other shot points were also captured. By considering the various shot points, different panel surface displacement patterns, directions, and failure modes might be obtained in the panels. This helped us to properly compare the responses of the panel at the specific target points, i.e., the moulded and nonmoulded shot points. In addition to the previous discussion considering impact at a nonmoulded area (shot points 3), Figure 12 shows the image sequences of an impacted panel with selected time at a moulded area (shot point 5). Here, only the two armour system panels that were not penetrated by all the shots, armour panels 8/4 and 8/8, were considered. In this particular shot point, armour panel 8/4 revealed higher surface displacement, which is located away from the penetrated contour area of the panel and mostly concentrated toward the four corners of the outer impacted regions. Even though the surface displacement of armour panel 8/8 shows a similar trend and directions to armour panel 8/4, it also revealed higher surface displacement intensity on the specified panel surface. Besides, the panel penetration sizes of armour system 8/8 were also found to be similar to armour panel 8/4 in their respective impact timing. For close observation and better discussion, the two tested but not penetrated armour panels, 8/8 and 8/4, were also presented in their conditions at the beginning and end of the impact period considering shot points 1, 3 and 5.

**Figure 13:**
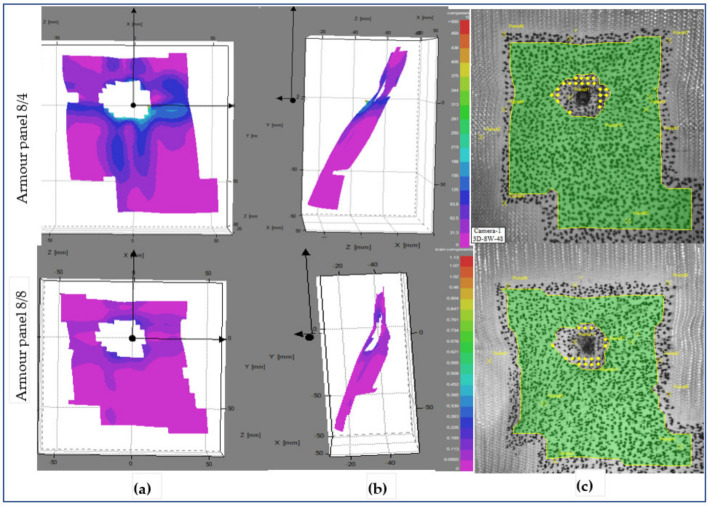
Surface displacement of frontal armour panel at impact times of 0.18 ms (**a**), (**b**) 3D frontal and side views of stereoscopic images respectively, and (**c**) frontal panel images at shot point 1.

**Figure 14:**
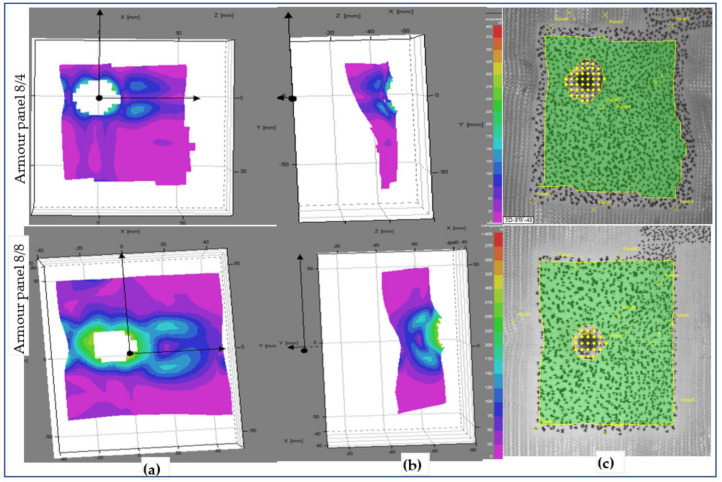
Surface displacement of frontal armour panel at impact times of 0.18 ms (**a**), (**b**), 3D frontal and side views of stereoscopic images, respectively, and (**c**) frontal panel images at shot point 3.

**Figure 15:**
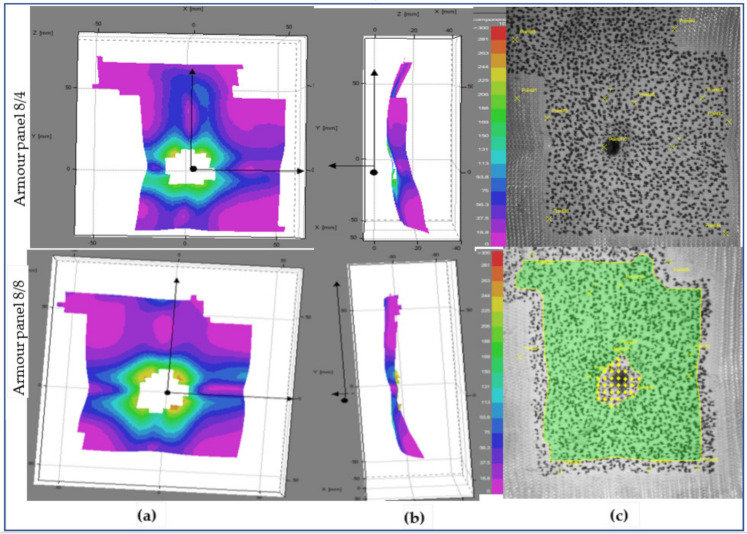
Surface displacement of frontal armour panel at impact times of 0.18 ms (**a**), (**b**), 3D frontal and side views of stereoscopic images, respectively, and (**c**) frontal panel images at shot point 5.

Figure 13, Figure 14 and Figure 15 show the surface displacements of the frontal planar and 3D steareoscopic and fabric panal images of the armour panels 8/8 and 8/4 with their local displacement at 0.18 ms after impact at shot points 1, 3 and 5, respectively. Based on the high-speed camera images, the surface displacement was initiated at t ≈ 0.180 ms after impact by the projectile wave for the mentioned tests. This is the reason why we consider t = 0.18 ms for the start of the impact to observe the different impacted armour systems. The armour systems were presented in the front and side views with the stereoscopic fabrics’ images. Figure 13 shows the deformed armour panels (8/4 and 8/8) with surface displacement at the instant (t = 0.18 ms) at shot 1. According to the observations, both armour systems show surface displacement on the panel; however, armour panel 8/4 involved a higher number of primary yarns in both directions as compared to armour panel 8/8 at the specified impact time.

This means that 8/8 needs much more time to detect the displacement than 8/4. However, as shown in the fabric images, the penetrated hole for both armour systems displays similar size and shape. Figure 13 shows the deformed panel (8/4 and 8/8) images with an instant time (t = 0.18 ms) after impact at shot point 3.

Unlike shot point 1, even though both armour systems involved larger primary yarn, armour panel 8/8 faces higher displacement values than 8/4 at shot point. The higher and instant involvements of the primary yarn after impact might be due to the high surface tension of the panel at this shot point. This result is because of the higher straightening (less undulation) of the yarn during the forming process in the particular impact area as compared to shot point 1. However, unlike the armour panel 8/8, armour system 8/4 shows similar displacement images in both shot points 1 and 3. Figure 15 also shows the tested armour panels (8/4 and 8/8) images with an instant time (t = 0.18 ms) after impact at shot point 5. At this particular shot point, both armour systems involved higher involvement of the primary regions with approximately equal displacement values around the impact regions. In general, armour panel 8/8 shows higher panel surface displacement at shot point 5 as compared to shot point 3 during the first few microseconds after impact (t = 0.18 ms). However, with the same impact timing, the panel revealed the lowest level of surface displacement at shot point 1. Armour panel 8/8 also faces higher displacement while waving propagation on the surface at shot point 5 at the start of the impact. On the contrary, the armour systems have seen smaller displacement intensity compared to shot 5 but approximately similar surface displacement intensity at shot point and 3 in the same impact timing.

**Figure 16:**
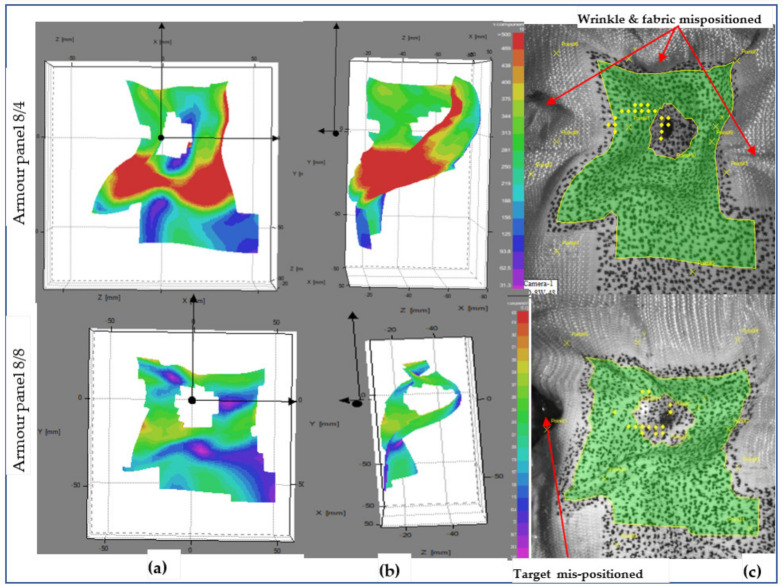
Surface displacement of frontal armour at impact times of 3.6 ms (**a**), (**b**), 3D frontal and side views of stereoscopic images, respectively, and (**c**) frontal fabric panel images at shot point 1.

**Figure 17:**
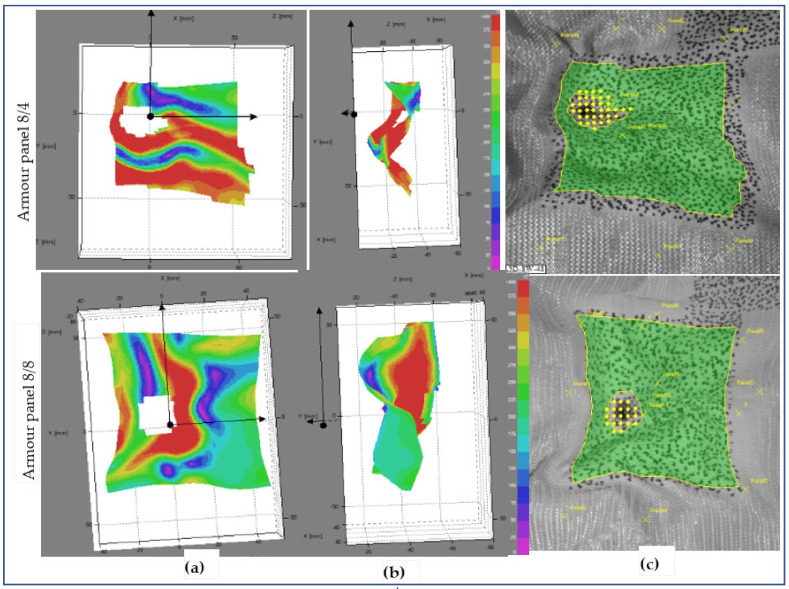
Surface displacement of frontal armour panel at impact times of 3.6 ms (**a**), (**b**), 3D frontal and side views of stereoscopic images, respectively, and (**c**) frontal panel images at shot point 3.

**Figure 18:**
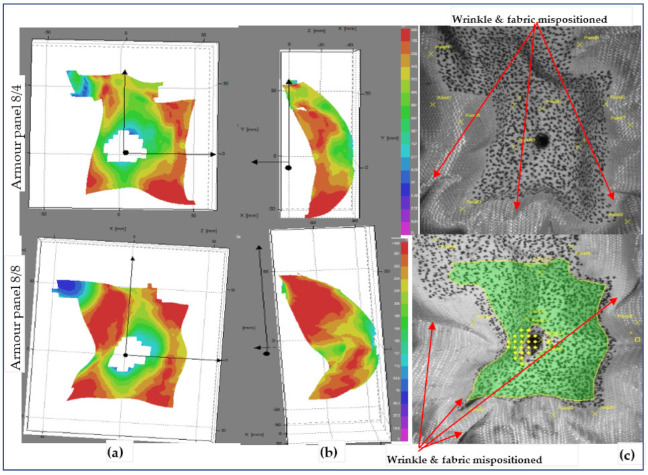
Surface displacement of frontal armour panel at impact times of 3.6 ms (**a**), (**b**), 3D frontal and side views of stereoscopic images, respectively, and (**c**) frontal panel images at shot point 5.

For both armour systems, a higher intensity of the surface displacement was achieved at shot 5 than the other shot points with the same impact time (t = 0.18 ms). Even the displacement values by the impact wave propagation were found to be higher at shot 3 as compared to shot point 1. This is due to the effect of the conditions of the shot areas during impact. Generally, the surface displacement values were more often observed at the shot points where the panel which faces higher tensioning of the yarns, including the moulded panel areas, than in the panel regions having higher undulations of yarn (nonmoulded regions). For a complete understanding of the impact phenomena, the armour systems’ surface displacement at the end of the impact process was also investigated with similar shot points, 1, 3 and 5. Figure 16, Figure 17 and Figure 18 show the surface displacement with 3D frontal and side views of the stereoscopic images and its panels for both 8/8 and 8/4 during the final microseconds of the impact (t = 3.6 ms) at shot point 1, 3 and 5, respectively. This phenomenon greatly helps us to observe the final states of the impacted armour systems not only in terms of the local surface displacement but also to see the global displacement and failure modes. Figure 16 illustrates the deformed armour panel (8/4 and 8/8) images with an instant time (t = 3.6 ms) after impact at shot point 1. According to the observations, armour panel 8/4 shows higher displacement surfaces just below the impacted location in the horizontal direction not only at the indicated postimpact time (t = 3.6 ms) but also for an extended impact duration.

Besides, global failures in the form of fabric wrinkling, shrinkage and mispositioning of the target in all directions were observed, as shown in the fabric images. On the contrary, armour system 8/8 revealed uniform distributions of the wave propagation with lower surface displacement values throughout the impacted local areas. In the global response, it also shows less fabric surface wrinkling but faces misposition of the target on the right sides Similarly, Figure 17 also displays the deformed armour panel (8/4 and 8/8) images with the time (t = 3.6 ms) after impact at shot point 3. Based on the stereoscopic image observations of the panel at the specific impact time and locations, both armour system types suffered from a huge amount of surface displacement as the impact wave propagated. However, each armour system panel tends to create displacements in a specific but different direction while resisting the ballistic impact. For example, in the armour panel 8/4, a higher surface displacement with different layers along the weft (horizontal) direction near the impact position and far edges of the impact positions were observed. Similarly, the other armour system, 8/8, also revealed a patterned surface displacement but was distorted along the warp (vertical) directions, mostly near to the impact points. Even though 8/8 shows higher values than 8/4, both armour systems show fewer values not only for global surface displacement but also for wrinkling and mispositions of the target at shot point 3 compared to shot point 1, as shown in fabric photographs. The local, global surface displacements and the other failure modes of the two armour systems were also observed at the impact position of the moulded area, shot point 5. This helps us to provide a better understanding of the effect of the impact position in terms of surface displacement and other failure modes.

Figure 18 shows the armour panels (8/4 and 8/8) deformation images at the time after impact (t = 3.6 ms) of shot point 5. As illustrated on the front faces of the surface displacement, 8/4 shows higher surface displacement around the far corners of the local impact points. Similarly, armour system 8/4 similarly faces surface displacement in similar positions to armour system 8/4 but with higher values. Both armour systems also face not only severe wrinkling and folding of the panel surface, but also the mispositioning of the panel toward the centre in all directions. Shot points 1 and 5 face higher global displacement in both armour systems as compared to shot point 3.

In the final remark, the global and local displacements at and around the impact regions were greatly affected not only by the type of the 3D warp interlock fabric variant but also by the impact shot points. Ballistic impact at the moulded areas (shot 5) brings not only higher local and global surface displacement but also the wrinkling and mispositioning of the panels toward the centre for both types of armour systems at the instant (t = 0.18 ms) and last (t = 3.6 ms) impact. Varient 8/4 shows higher surface local and global displacements at shot points 1 and 3, but less global displacement at shot point 3.

## 4. Conclusions

Different studies have found 3D woven fabrics as an efficient structure for various applications, particularly for flexible and soft body armour designs, due to their good impact and formability performances. However, such fabrics should be investigated with various parameters and their different impact behaviours should be understood before their application in armour systems. The current research has tested armour panel systems made with different 3D warp interlock fabric variants against the NIJ Level-IIIA standard and studied their different postmortem behaviours. Based on the results, armour panel systems made of fabric variant 8/0 revealed perforation at two different shot points, whereas armour panels made with fabric variant 4/8 were completely perforated in all shot points. On the contrary, armour panel systems made of fabric variants 8/8 and 8/4 did not face any perforation at all. The postmortem analysis of the projectile deformations and the number of panels involved in the armour system to stop the projectile were also influenced by the warp yarn ratios of the 3D warp interlock fabrics. For example, the armour panels made of fabric variants 8/4 and 8/8 revealed the same minimum number of panels responsible for halting the projectile in the majority of the shot points. Those armour systems also involved only five panels at shot points 1 and 2 to arrest the projectile. Besides, only panels four and two were accountable for arresting the projectile by the two armour panels at shot points three and five, respectively. Armour panel system made of fabric variant 8/8 uses a lesser number of panels to stop the projectile at shot points four and six as compared to armour made of fabric variant 8/4. Moreover, based on images from the high-speed camera, the global and local displacement values were also greatly affected not only by the type of the 3D warp interlock fabric variant but also by the impact shot points. Armour panel made of fabric variant 8/4 shows higher surface local and global displacements at shot point one and three, but less global displacement at shot points three. Moreover, the armour panel made of fabric variant 8/8 shows less global and local displacements at shot points three and one, respectively compared with other impact points. Ballistic impact at the moulded areas (shot five) brings not only higher local and global surface displacement but also wrinkling and mispositioned of the panels toward the centre for both types of the armour at the instant (t = 0.18 ms) and last (t = 3.6 ms) impact timing. The current research findings are enlightened for further engineering’s of 3D woven fabric for seamless women impact protective clothing.

## Data Availability

The data presented in this study are available on request from the corresponding authors.
